# Comparison of Ramosetron with Palonosetron for Prevention of Postoperative Nausea and Vomiting in Patients Receiving Opioid-Based Intravenous Patient-Controlled Analgesia after Gynecological Laparoscopy

**DOI:** 10.1155/2017/9341738

**Published:** 2017-03-05

**Authors:** Eun Jin Ahn, Geun Joo Choi, Hyun Kang, Chong Wha Baek, Yong Hun Jung, Young Cheol Woo

**Affiliations:** ^1^Department of Anesthesiology and Pain Medicine, Inje University Seoul Paik Hospital, Seoul, Republic of Korea; ^2^Department of Anesthesiology and Pain Medicine, Chung-Ang University College of Medicine, Seoul, Republic of Korea

## Abstract

We aimed to compare the effects of ramosetron and palonosetron in the prevention of postoperative nausea and vomiting (PONV) in patients that received opioid-based intravenous patient-controlled analgesia (IV-PCA) after gynecological laparoscopy. We reviewed the electronic medical records of 755 adults. Patients were classified into two groups, ramosetron (group R, *n* = 589) versus palonosetron (group P, *n* = 166). Based on their confounding factors, 152 subjects in each group were selected after the implementation of propensity score matching. The overall incidence of PONV at postoperative day (POD) 0 was lower in group R compared to group P (26.9% versus 36.8%; *P* = 0.043). The severity of nausea was lower in group R than in group P on postoperative day (POD) 0 (*P* = 0.012). Also, the complete responder proportion of patients was significantly higher in group R compared to that in group P on POD 0 (*P* = 0.043). In conclusion, ramosetron showed a greater efficacy in the prevention of postoperative nausea at POD 0 compared to palonosetron in patients after gynecological laparoscopy.

## 1. Introduction

Postoperative nausea and vomiting (PONV) is an unpleasant and distressing complication after anesthesia and surgery [1] which can prolong the hospital stay, increase healthcare costs, and decrease patient satisfaction [2]. Notably, the incidence of PONV after gynecological laparoscopy is reported to be nearly 80% [3]. PONV may also be induced by opioid analgesics, which are widely used for patient-controlled analgesia (PCA) for pain control during the early postoperative phase. Therefore, PONV after gynecological laparoscopy is an important clinical problem to be solved.

Among numerous antiemetics which have been studied to prevent and treat PONV, selective serotonin 5-hydroxytryptamine type 3 (5-HT3) receptor antagonists have shown efficacy in the prophylaxis and treatment of PONV [4]. Ramosetron and palonosetron are recent developments in selective 5-HT3 receptor antagonists. Ramosetron is a 5-HT3 antagonist, which exhibits higher receptor affinity and a slower dissociation rate compared to older agents in its class [5, 6]. Palonosetron is a second-generation 5-HT3 receptor antagonist with an even higher binding affinity and a prolonged half-life (mean 40 h) [7].

The aim of this retrospective study was to compare the efficacy of ramosetron and palonosetron in the prevention of PONV in patients that received opioid-based IV-PCA after gynecological laparoscopy.

## 2. Materials and Methods

### 2.1. Study Design

After Institutional Review Board approval (C2016048 [1791]), we reviewed the medical records of 826 adults that received fentanyl-based IV-PCA after gynecological laparoscopy at Chung-Ang University Hospital between January 1, 2010, and December 31, 2016. Patient information was correctly anonymized and identified prior to analysis. Informed consent was waived for this study as it was not required. Only patients who were administered a single prophylactic antiemetic with a 5-HT3 receptor antagonist postoperatively were included in this study. We classified the patients into two groups (group R = ramosetron; group P = palonosetron), based on the use of antiemetics. The patients were excluded if reoperations were performed or more than one antiemetic was administered.

In our institution, standardized IV-PCA protocol of the department of anesthesiology and pain medicine was applied to all patients. The correct dosages of fentanyl, ketorolac, or nefopam were established according to the department of surgery's concerns and the expected intensity of pain (mild, moderate, or severe) after each type of surgery. For laparoscopic gynecologic minor surgery in which mild postoperative pain was expected, fentanyl 15 mcg/kg, ketorolac 180 mg (or nefopam 120 mg), and antiemetics (ramosetron 0.3 mg or palonosetron 0.25 mg) were added to normal saline to make a 100 cc solution. The preset continuous infusion rate of IV-PCA was 1 cc/hr, bolus dose 1 cc, and a lockout interval 15 minutes. For laparoscopic gynecologic major surgery in which moderate or severe postoperative pain was expected, fentanyl 25 mcg/kg, ketorolac 180 mg (or nefopam 120 mg), and antiemetics (ramosetron 0.3 mg or palonosetron 0.25 mg) were added to a normal saline to make a 100 cc solution. The setting of IV-PCA was same as minor surgery. PCA was started just after induction of anesthesia and ramosetron 0.3 mg or palonosetron 0.25 mg was administered just before end of surgery.

### 2.2. Data Collection

Relationships between demographic and perioperative variables and the factors of PONV were noted. Age, height, weight, history of smoking, PONV, type of anesthetic used (Desflurane versus Sevoflurane), use of premedication, N_2_O, and remifentanil, operation time, dose of analgesics used in PCA, and the use of Acupan® were factors involved in the collection of data. As postoperative variables, dizziness, headache, and the amount of vomiting were measured. Additional variables included the potential requirement of rescue antiemetics and CR (complete responder) status. Complete response was defined as “the absence of nausea and vomiting and non-requirement of antiemetic medication.”

The nurse, dedicated to the management of patients with IV-PCA, evaluated the severity of pain using a 10-point visual analogue scale (VAS) and the severity of nausea using a numerical rating scale (none = 0; mild = 1; moderate = 2; severe = 3; worst imaginable = 4). All of the aforementioned variables were measured on POD 0 and POD 1.

The nurse only undertook tasks related to IV-PCA, and she made the rounds at least once a day to investigate issues related to IV-PCA, including pain and PONV. She had 5 years' clinical experience, and she collected data after being trained in the standardized protocols of pain and PONV investigation.

The primary endpoint was the overall incidence of PONV at POD 0. The complete responders were also calculated by the overall incidence of PONV. The severity of nausea and requirement of rescue antiemetics were secondary outcomes in this study.

### 2.3. Statistics

Propensity score matching was performed to match patients from each group in a 1 : 1 ratio and reduce potential confounding variables [8]. Given that this was a retrospective cohort study and not a randomized trial, it was necessary to achieve comparability of the ramosetron group and palonosetron group with regard to potential confounding variables by nonrandom assignment or unbalanced covariates. The propensity score was calculated by logistic regression analysis using the following covariates: age, height, weight, history of smoking, PONV, type of anesthetic used (Desflurane versus Sevoflurane), use of premedication, N_2_O, and remifentanil, operation time, dose of analgesics used in PCA, and the use of Acupan [9]. After calculating the propensity scores, we chose the nearest available match in order to pair each participant between the groups based on the propensity score similarities. To assess the achieved balance between the matched groups, we tested for the standardized differences for each baseline covariate. Standardized difference is the difference in the means between the two groups expressed in units of standard deviation [10]. A value of less than 20% is considered to indicate an adequate balance and, therefore, good comparability between the groups.

Before matching, baseline demographics and clinical characteristics were summarized using descriptive statistics. For continuous variables, data were presented as the mean-standard deviation, and groups were compared using the unpaired *t*-test. The descriptive variables were analyzed by either a Chi-square test or Fisher's exact test, as determined appropriate.

Propensity matched continuous variables are shown as the mean-standard deviation and categorical variables are shown as absolute numbers (percentages). Statistical differences between the groups were tested with independent *t*-tests and McNemar's test. A *p* value < 0.05 was considered statistically significant. All statistical analyses were performed using the SPSS software suite (IBM Corp., Armonk, NY, USA).

## 3. Results

**Figure 1:**
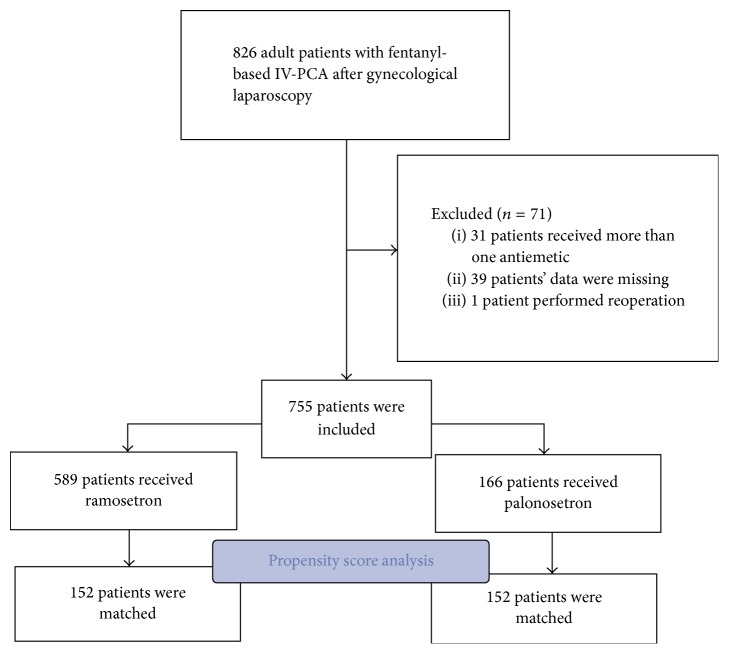
Flow diagram.

The basic demographics and clinical characteristics of the patient population are detailed in Table 1. Among 826 adults that received fentanyl-based IV-PCA after laparoscopic gynecological surgery at Chung-Ang University Hospital between January 1, 2010, and December 31, 2016, 71 patients were excluded from our study due to missing data (*n* = 39), reoperation (*n* = 1), or the use of more than one antiemetic (*n* = 31). Therefore, a total of 755 patients were included in this study, with 589 in group R and 166 in group P (Figure 1).

### 3.1. Ramosetron versus Palonosetron in the Overall Series

Of 14 individual and composite predictors of confounding variables, 5 had poor standardized difference scores prior to the propensity score matching. Compared with group R, subjects in group P received a higher dose of fentanyl in PCA (1041.23 ± 145.94 mcg versus 1102.99 ± 195.97, *P* < 0.001; Table 1). In addition, subjects in group P showed a lower incidence of receiving premedication drugs (365 [63.9%] versus 69 [38.5%], *P* < 0.001; Table 1), N_2_O (481 [84.2%] versus 130 [72.6%], *P* < 0.001; Table 1), preintubation opioids (425 [74.4%] versus 111 [62.0%], *P* = 0.001; Table 1), and Acupan (289 [50.6%] versus 54 [30.2%], *P* < 0.001; Table 1).

All variables including pain VAS, nausea NRS, CR, amount of vomiting, dizziness, and headache on POD 0 and POD 1 showed no significant differences between group R and group P (Table 2).

### 3.2. Ramosetron versus Palonosetron according to Propensity Score Analysis

After the propensity score analysis, 152 patients remained (Figure 1). All 14 confounding variables had acceptable standardized difference scores (<20%) indicating that the matching procedure was efficient in creating balance between the two groups. After adjusting the propensity score analysis, the overall incidence of PONV at POD 0 was lower in group R compared to group P (26.9% versus 36.8%; *P* = 0.043). The nausea NRS on POD 0 was reported as higher in group P compared to group R (0.15 ± 0.44 versus 0.34 ± 0.79; *P* = 0.012). Also, CR status on POD 0 was significantly higher in group R compared to group P (73% versus 63.2%; *P* = 0.043). However, no significant difference was observed in nausea NRS and CR status on POD 1, pain VAS, rescue antiemetics, amount of vomiting, dizziness, or headache on POD 1 and POD 2 between group R and group P (Table 3).

## 4. Discussion

The etiology of PONV remains unclear but involves anesthetic, surgical, patient, and PCA factors. Well-known patient specific risk factors include female gender, nonsmoking, and history of motion sickness or PONV, whereas nonspecific factors involve the use of postoperative opioids and the type of surgery performed, such as laparoscopy [11, 12]. In our present study, all patients exhibited at least three risk factors including female gender, postoperative opioid use, and laparoscopy. The results have shown that ramosetron was more effective in the prevention of postoperative nausea compared to palonosetron. However, there was no significant difference in the prevention of vomiting between both drugs.

The area postrema, or vomiting center, controls and coordinates nausea and vomiting and is located in the lateral reticular formation of the medulla. This center receives various inputs from receptors in the gastrointestinal tract, peripheral pain receptors, the nucleus solitaries, the vestibular system, the cerebral cortex, and the chemoreceptor trigger zone [13]. The high incidence of PONV after laparoscopy is explained by the compression of the gastrointestinal mucosa by the surgical pneumoperitoneum which may induce intestinal ischemia and thus trigger a serotonin release leading to PONV [14]. The central action of carbon dioxide (CO_2_), stretching of the peritoneum and diaphragm, and increased blood pressure in the peritoneal cavity after CO_2_ insufflation are considered to provoke PONV by reducing blood flow [15, 16]. Therefore, a variety of serotonin receptor (5-HT3) antagonists with a similar mechanism (selective or competitive binding to 5-HT3 receptors) have been used to manage PONV [17]. In this study, we compared two antiemetics, ramosetron and palonosetron, for the prevention of PONV after gynecological laparoscopy. Similar to the results of previous studies, ramosetron was superior to palonosetron in preventing postoperative nausea [13, 18]. However, discrepancies exist in a number of studies that compared ramosetron and palonosetron. The studies of Lee showed no significant differences between ramosetron and palonosetron in the incidence of PONV in patients who underwent gynecological laparoscopy [19, 20].

Our study showed a significant difference between ramosetron and palonosetron for overall incidence of PONV and CR rate on POD 0. Also, postoperative nausea NRS scores on POD 0 were significantly higher in the ramosetron group than in the palonosetron group on POD 0. However, there are a number of studies which reported pharmacologic difference between the ramosetron and palonosetron. In the study of Swaika, ramosetron was more effective than palonosetron in the early postoperative period (0–2 h) [13]. But, in the time periods of 2–6 h and 6–24 h, there was no statistically significant difference between both groups [13]. Therefore, further studies would be needed to find the pharmacologic influence in this result.

There were limitations in this study. First, although the dose of antiemetics administered may have affected the results, we were unable to evaluate the dose of antiemetics administered. Second, as previously stated, we considered the possibility of missing data due to the retrospective design and the extended time span between the first and the last included case. Finally, this was a single center study in Korea; generalized results may not be applicable to patients in other countries. In spite of the retrospective design, we highlight the strengths of our study by noting the substantial amount of clinical data that was analyzed and the performance of propensity score matching to avoid confounding selection bias.

In conclusion, ramosetron has shown greater efficacy for the prevention of PONV at POD 0 in comparison to palonosetron in patients that underwent laparoscopic gynecological surgery.

## Figures and Tables

**Table 1:** Patient characteristics in total and matched cohorts.

Characteristic	Total set				Matched set			
	Group R (*n* = 589)	Group P (*n* = 166)	STD (%)	*P* value	Group R (*n* = 152)	Group P (*n* = 152)	STD (%)	*P* value
Age	42.68 ± 19.96	39.64 ± 12.66	−16.34	0.056	39.95 ± 12.82	39.36 ± 14.42	−4.32	0.675
Height	159.14 ± 6.93	159.68 ± 9.21	7.21	0.418	159.23 ± 5.91	159.45 ± 8.32	3.05	0.735
Weight	57.66 ± 9.15	57.46 ± 9.74	−2.15	0.810	57.60 ± 9.03	57.51 ± 8.32	−1.04	0.923
OP time	139.21 ± 111.01	156.36 ± 165.98	13.7	0.113	136.62 ± 93.18	152.51 ± 165.53	11.83	0.263
PCA fentanyl (mcg)	1041.23 ± 145.94	1102.99 ± 195.97	39.02	<0.001	1064.63 ± 191.24	1081.55 ± 191.49	8.84	0.412
Smoking	40 (7.0)	14 (7.8)	10.81	0.712	8 (5.3)	8 (5.3)	0	1.000
PONV history	30 (5.3)	13 (7.3)	31.75	0.313	11 (7.2)	10 (6.6)	−8.33	1.000
Premedication	365 (63.9)	69 (38.5)	49.61	<0.001	69 (45.4)	63 (41.4)	−8.81	0.135
Sevoflurane	203 (35.6)	72 (40.2)	12.14	0.258	56 (36.8)	65 (42.8)	16.30	0.336
Desflurane	368 (64.4)	107 (59.8)	7.41	0.258	96 (63.2)	97 (63.8)	0.95	0.981
N_2_O	481 (84.2)	130 (72.6)	14.80	<0.001	122 (80.3)	118 (77.6)	−3.36	0.418
Preintubation opioid	425 (74.4)	111 (62.0)	18.18	0.001	101 (66.4)	99 (65.1)	−1.96	0.838
Remifentanil	42 (7.4)	15 (8.4)	12.66	0.652	15 (9.9)	12 (7.9)	−20.20	0.664
Acupan	289 (50.6)	54 (30.2)	50.50	<0.001	58 (38.2)	52 (34.2)	−10.47	0.504

Values are expressed as mean ± SD, absolute number (percentages), or absolute number. OP: operation, STD: standardized difference, PONV: postoperative nausea and vomiting, and PCA: patient-controlled analgesia.

**Table 2:** Perioperative variables before matching.

	Group R (*n* = 589)	Group S (*n* = 166)	STD (%)	*P* value
Pain VAS at day 0	6.29 ± 1.93	6.23 ± 172	−0.07	0.346
Pain VAS at day 1	3.07 ± 1.55	2.86 ± 1.37	−13.89	0.108
Nausea NRS at day 0	0.25 ± 0.65	0.35 ± 0.82	14.48	0.111
Nausea NRS at day 1	0.06 ± 0.27	0.03 ± 0.26	−11.2	0.212
Rescue antiemetics at day 0	75 (13.1)	28 (15.6)	17.42	0.395
Rescue antiemetics at day 1	33 (5.6)	10 (6.0)	6.90	0.835
PONV at day 0	169 (28.7)	59 (35.5)	−10.01	0.090
PONV at day 1	67 (11.4)	23 (13.8)	2.86	0.651
CR at day 0	420 (71.3)	107 (64.5)	10.01	0.090
CR at day 1	522 (88.6)	143 (86.1)	−2.86	0.651
Number of vomiting instances at day 0	0.05 ± 0.43	0.03 ± 0.26	−5.01	0.544
Number of vomiting instances at day 1	0.00 ± 0.59	0.01 ± 0.09	1.91	0.429
Dizziness at day 0	14 (2.5)	2 (1.1)	77.78	0.382
Dizziness at day 1	13 (2.3)	3 (1.7)	30	0.774
Headache at day 0	1 (0.2)	1 (0.6)	100	0.421
Headache at day 1	3 (0.5)	0 (0.0)	200	1.000

Values are expressed as mean ± SD, absolute number (percentages), or absolute number. STD: standardized difference, VAS: visual analogue scale, NRS: numerical rating scale, and CR: complete responder.

**Table 3:** Perioperative variables after matching.

	Group R (*n* = 152)	Group P (*n* = 152)	STD (%)	*P* value
Pain VAS at day 0	6.17 ± 1.87	6.17 ± 1.79	0	0.977
Pain VAS at day 1	2.99 ± 1.58	2.86 ± 1.41	−8.68	0.443
Nausea NRS at day 0	0.15 ± 0.44	0.34 ± 0.79	29.71	0.012^*∗*^
Nausea NRS at day 1	0.05 ± 0.25	0.09 ± 0.35	13.15	0.275
Rescue antiemetics at day 0	16 (10.5)	27 (17.8)	69.52	0.080
Rescue antiemetics at day 1	9 (5.9)	8 (5.3)	−10.17	0.803
PONV at day 0	41 (26.9)	56 (36.8)	13.42	0.043^*∗*^
PONV at day 1	15 (9.8)	21 (13.8)	4.33	0.287
CR at day 0	111 (73.0)	96 (63.2)	−13.42	0.043^*∗*^
CR at day 1	137 (90.1)	131 (86.2)	−4.33	0.287
Number of vomiting instances at day 0	0.03 ± 0.27	0.03 ± 0.27	0	1.000
Number of vomiting instances at day 1	0.00 ± 0.00	0.01 ± 0.08	17.68	0.319
Dizziness at day 0	2 (1.3)	0 (0.0)	−100	1.000
Dizziness at day 1	2 (1.3)	0 (0.0)	−100	1.000
Headache at day 0	0 (0.0)	0 (0.0)		NA
Headache at day 1	0 (0.0)	0 (0.0)		NA

Values are expressed as mean ± SD, absolute number (percentages), or absolute number. STD: standardized difference, VAS: visual analogue scale, NRS: numerical rating scale, and CR: complete responder. ^*∗*^*P* < 0.05 between group comparison.
